# Golden thistle (*Scolymus hispanicus* L.) hydromethanolic extracts ameliorated glucose absorption and inflammatory markers in vitro

**DOI:** 10.1002/fsn3.3716

**Published:** 2023-10-08

**Authors:** Cansu Ozel‐Tasci, Sukru Gulec

**Affiliations:** ^1^ Department of Food Engineering, Molecular Nutrition and Cell Physiology Laboratory Izmir Institute of Technology Urla Izmir Turkey

**Keywords:** antidiabetic activity, colonic inflammation, golden thistle, phenolic bioavailability, *Scolymus hispanicus* L., systemic inflammation

## Abstract

Golden thistle (GT, *Scolymus hispanicus* L.) is an edible plant native to the Mediterranean. Several activities have been reported for the GT, as it is used for traditional medicinal purposes in some cultures. In this study, we aimed to investigate the effects of GT crude extract on phenolic bioavailability, antidiabetic, and anti‐inflammatory activities by using colonic epithelium (CaCo‐2) and murine macrophage (RAW 264.7) cell lines. The CaCo‐2 cells were grown on the bicameral membrane system for intestinal bioavailability and glucose efflux. Lipopolysaccharide (LPS, 0.5 μg/mL) was used to induce systemic inflammation on RAW 264.7. The inflammatory medium of RAW 264.7 cells was given to Caco‐2 cells to mimic colonic inflammation. Our results showed that 5‐*o*‐caffeoylquinic acid had an apparent permeability of (1.82 ± 0.07) × 10^−6^ cm/s after 6 h. The extract lowered the glucose efflux by 39.4%–42.6%, in addition to the reductions in relative GLUT2 mRNA expressions by 49%–66% in pre‐ and co‐treatments (*p* < .05). Decreases in systemic inflammation markers of nitric oxide, tumor necrosis factor‐alpha, and interleukin‐6 (IL‐6) were also detected in 30%–45% range after pre‐treatments with the GT extract (*p* < .05). Lastly, colonic inflammation markers of IL‐6 and IL‐8 were reduced by 8.7%–19.5% as a result of GT pre‐treatments (*p* < .05). Thus, an in vitro investigation of GT extract revealed promising results on antidiabetic and anti‐inflammatory activities.

## Graphical abstract

A screening study was conducted on golden thistle (*Scolymus hispanicus* L.) hydromethanolic extract using CaCo‐2 and RAW 264.7 cell lines in vitro. The extract had relatively lower chlorogenic acid bioavailability but significantly reduced glucose efflux and relative GLUT2 mRNA expression. The results of two inflammation models also revealed potential preventive effects for various inflammation markers of NO, TNF‐α, and IL‐6 in systemic, IL‐6, and IL‐8 in colonic inflammation.

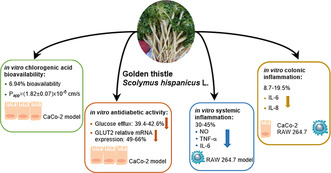

## 1 INTRODUCTION

The Mediterranean lifestyle has been recommended by the nutritionists due to its preventive effects and healthy ingredients (Burlingame & Dernini, 2011; Sofi et al., 2013; Trichopoulou & Vasilopoulou, 2000). In addition to those ingredients, the Mediterranean countries are also known for their wild edible plant consumption for culinary and medicinal purposes (González‐Tejero et al., 2008). The golden thistle plant (GT, *Scolymus hispanicus* L.) of the Asteraceae family is an important plant in that context. It has been consumed in the cuisines of Turkey, Greece, Spain, Portugal, Italy, Cyprus, and Morocco (Della et al., 2006; Ertuğ, 2004; Lentini & Venza, 2007; Pardo‐De‐Santayana et al., 2005; Powell et al., 2014; Sergio et al., 2023). In traditional medicine, this plant has been used against ulcers, eye infections, intestinal diseases, and Malta fever (Berdja et al., 2021; Ugurlu & Secmen, 2008). Moreover, plant infusions or extracts have been shown to inhibit mammalian glycosidases in vitro (Marmouzi et al., 2017) or to lower body weights and fasting blood glucose levels in animal models (Berdja et al., 2021; Özkol et al., 2012), indicating GT has antidiabetic potential. Asteraceae plants are also associated with antioxidant and anti‐inflammatory effects which make them candidates as raw materials in functional food and nutraceutical developments (Awwad et al., 2020; Mohanta et al., 2023). However, the studies on GT are limited. Moreover, bioavailability is an important parameter for phytochemical functionality. In literature, the bioavailability of natural phytochemicals is considered low by many researchers (Ahmad et al., 2022; Chen et al., 2022; Santhiravel et al., 2022). Hence, for crude extracts, it might be important to investigate the bioavailable portion of the phytochemicals of GT.

Considering all these traditional usages and scientific reports, we aimed to conduct a screening study for the cultivated golden thistle extract (GTE) in terms of phenolic bioavailability, as well as antidiabetic and anti‐inflammatory activities by utilizing in vitro cell culture models.

## 2 MATERIALS AND METHODS

### 2.1 Materials and chemicals

The golden thistle samples utilized in our study have been bred, officially registered, and kept in National Seed Gene Bank of the Turkish Republic Ministry of Agriculture and Forestry with the voucher specimen IZ herbarium UK 08.06.2018 0101. The plants were cultivated in the agricultural fields in Menemen, İzmir province, by agricultural engineers responsible for medicinal plants. The plant parts as aerial, root bark (RB), and root internal (RI) were obtained individually from Turkish General Directorate of Agricultural and Research Policies (TAGEM) in dry powdered form. To achieve this, individual plant parts were dried at 50°C immediately after harvest. Then, the dried samples were ground into powdered form.

Minimum essential medium (MEM, M4655), RPMI‐1640 medium (R8758), Penicillin Streptomycin (P4333), non‐essential amino acids (M7145), sodium pyruvate (S8636), 1‐(4,5‐Dimethylthiazol‐2‐yl)‐3,5‐diphenylformazan (MTT, M2003), and lipopolysaccharide from *Escherichia coli* (L4516), pepsin, guar, pancreatin, amyloglucosidase, invertase, Hank's balanced salt solution (HBSS), chlorogenic acid standard (PHL89175), and Trizol® reagent were purchased from Sigma‐Aldrich. Heat inactivated FBS (10500) was from Gibco™ (Thermo Fisher Scientific). Six‐well inserts for bioavailability (Transwell®), and 12‐well insert membranes for the glucose efflux studies (Transwell®) were of Corning. cDNA synthesis kit (EQ003), SYBR‐Green mix (EQ007) were from ELK Biotech. Primers of mRNA expression studies (Invitrogen™) were purchased from Lifetech.

Mouse tumor necrosis factor alpha (TNF‐α, EK0527), mouse interleukin 6 (IL‐6, EK0411), human IL‐6 (EK0410), human IL‐8 (EK0413) enzyme‐linked immunosorbent assay (ELISA) kits were purchased from Boster Biological. Unless stated otherwise, all solvents and chemicals used in the extraction and experiments were analytical grade.

### 2.2 Extraction

The extraction procedure was conducted from the study of Malami et al. (2016) with some additional modifications. The extracts were obtained individually. Ten grams powder for each sample was first extracted with 200 mL 90% methanol for 16 h with continuous stirring. The extraction temperature was 40°C for aerial and 80°C for root bark and root internal parts. Then, the samples were centrifuged at 700 *g* for 5 min (Centric 322A, Tehtnica), and the supernatants were collected. The pellets were re‐extracted with an additional 200 mL MilliQ® water for 4 h at their corresponding temperatures. After centrifuging the water extracts at the same conditions again, the supernatants were collected and mixed with methanol fractions. The methanol in the extract was evaporated at 40°C under vacuum, and the remaining extracts were freeze‐dried. During the experiments, the individual freeze‐dried extracts were dissolved in appropriate cell culture mediums or buffers, mixed in equal volumes as one sample, and called GTE.

### 2.3 Phytochemical screening and determination of antiradical activity

The total phenolic and flavonoid contents of each extract were determined according to Rufino et al. (2010) and Marmouzi et al. (2017) without any modifications, respectively. Gallic acid was used as the standard of total phenolic content determination, whereas for flavonoid content assay, both catechin and rutin were utilized as standards.

Trolox equivalent antioxidant capacity (TEAC), DPPH radical scavenging activity, and ferric reducing antioxidant power (FRAP) were conducted by using Re et al. (1999), Martinez‐Morales et al. (2020), and Benzie and Strain (1996) procedures for each assay, respectively. Trolox and ascorbic acid were the utilized standards for the corresponding method.

### 2.4 Cell maintaining and cytotoxicity

CaCo‐2 (HTB‐37™) and RAW 264.7 (TIB‐71™) cells used in the study were purchased from American Tissue Culture Collection (ATCC®, Virginia). CaCo‐2 cells were maintained in MEM with 15% FBS, 1% non‐essential amino acid solution, 1% sodium pyruvate, and 1% penicillin–streptomycin solution (passage numbers: 30–40), whereas RAW 264.7 cells were maintained in RPMI medium with 10% FBS and 1% penicillin–streptomycin solution (passage numbers: 15–25). The cells were maintained and experimented at a 37°C incubator containing 5% CO_2_.

Before treatments for the corresponding assay, a cytotoxicity test was applied to determine the non‐toxic concentrations. Briefly, the cells were seeded in 4000 cell/well density on a 96‐well plate and incubated at the maintaining conditions overnight for attachment (Bonnefont et al., 1998). The following day, GTE treatments were given to the cells for 6–24 h, followed by an MTT viability assay of Morgan (1998) at 570 nm (ThermoskanGO, Thermo Scientific).

### 2.5 In vitro bioavailability model

The in vitro bioavailability model was conducted with CaCo‐2 cell line on six‐well transmembrane inserts according to the related literature reports (Hithamani et al., 2017; Konishi & Shimizu, 2003). The cells were seeded in 1 × 10^5^ cell/well density on the apical side of transmembrane inserts and incubated for attachment. The cell culture medium was changed every other day until the experiment. When the cells reached 100% confluency, a post‐confluency period was counted for 21 days. Then, the transepithelial electrical resistance (TEER) was measured (EVOM^2^, World Precision Instruments), and the TEER value of the membranes at least 250 Ω cm^2^ was included in the experiments. During the experiment, 1 mg/mL GTE was prepared in HBSS and given to the apical side of the transmembrane inserts. The basolateral sides contained HBSS without any additional compounds. Samples were taken from the basolateral side periodically during the experiment for 6 h and analyzed with high‐pressure liquid chromatography (HPLC). For that purpose, the same procedure from Uncu and Ozen (2015) was used to determine the phenolic acids in the GTE both before and after bioavailability studies, without any modifications.

The results obtained from the bioavailability assay were calculated as apparent permeability (*P*_app_) according to the study of Zhou et al. (2015) given in Equation 1:

(1) $$P_{\mathrm{app}}=\frac{\left(\frac{dC}{dt}\right)\times V}{A\times C_{0}}$$

in which *P*_app_ is the apparent permeability in cm/s, d*C*/d*t* represents the concentration gradient with respect to time (ppm/min), *V* is the volume of the basolateral side in mL, *A* is the insert membrane area in cm^2^, and *C*_0_ is the initial concentration applied to the apical side of the insert membrane in ppm.

### 2.6 In vitro digestion

To investigate the effect of the extract on starch digestibility, an in vitro digestion method was applied using 1% of starch solution with our previous work (Ozel‐Tasci et al., 2020). The experimental groups were as follows: A GTE sample in which no enzyme was used in the digestion procedure (GTE_blank_), a solely digested GTE, a control group of 1% starch solution (Starch), and a 1% starch solution to contain 1 mg/mL GTE (Starch + GTE) at the end of the digestion procedure. Free glucose, either present or released after the digestion procedure, was analyzed with HPLC. For glucose determination, the method from Surek and Buyukkileci (2017) was applied without any modifications.

### 2.7 Glucose efflux model

A similar procedure to the bioavailability study was conducted to measure the glucose efflux of the CaCo‐2 cells when they were treated with the GTE extract with slight modifications. Same cell seeding and post‐confluency maintaining, as well as the TEER measurement, were also included in this part of the study using 12‐well insert membranes. For the glucose efflux study, 25 mM glucose‐containing Krebs buffer was used instead of HBSS medium on the apical side of the transmembrane inserts. There were three experimental groups. The first was the control group, in which the cells were given only 25 mM glucose in Krebs buffer. In the second group, CaCo‐2 cells were pre‐treated with 1 mg/mL of GTE for 2 h and discarded before glucose addition. In the last of the experimental groups, the GTE at 1 mg/mL concentration and 25 mM glucose containing Krebs transport buffer was introduced to the apical side at the same time and called as co‐treated group. In all, the basolateral part consisted of only Krebs buffer without glucose. Like the bioavailability study, samples were taken from the basolateral side periodically, and the glucose contents were measured with HPLC (Surek & Buyukkileci, 2017).

### 2.8 RT‐qPCR

The cells that were treated with GTE or the control group in the glucose efflux study were tested to determine the mRNA expression changes in a common glucose transporter, GLUT2. Total mRNA isolation using Trizol®, cDNA synthesis, and RT‐qPCR were applied to the same cells of in vitro glucose efflux assays according to the instructions of corresponding manufacturers. Both primers and RT‐qPCR protocol were applied according to the descriptions of Boztepe and Gulec (2018). Human cyclophilin A was used as the housekeeping gene, and the determined mRNA expressions were normalized to this housekeeping gene. The data were analyzed with the $2^{-∆∆C_{t}}$ method, and the results were given as fold changes. The primers used for the genes are as follows: GLUT2 Forward: CTCTCCTTGCTCCTCCTCCT and Reverse: TTGGGAGTCCTGTCAATTCC; Cyclophilin A Forward: TACGGGTCCTGGCATCTTG and Reverse: CGAGTTGTCCACAGTCAGCA.

### 2.9 Systemic inflammation model

RAW 264.7 cells were used to determine the effects of the GTE on systemic inflammation as in the study of Chun et al. (2006) with some modifications. The cells were seeded in 1 × 10^6^ cell/well in 12‐well cell culture plates. After an overnight attachment, the cells were treated with a starving medium containing 3% FBS to eliminate the effects of FBS on cytokine release for 6 h. Then, 50 and 500 μg/mL of GTE were given for 2 h as pre‐treatments. At the end of the pre‐treatment period, the extract‐containing mediums were discarded and 0.5 μg/mL lipopolysaccharide (LPS) containing medium was introduced to the pre‐treated cells to induce inflammation. Inflammation was constituted for 12 h, and mediums were collected from each experimental group at the end of the period. According to the kit instructions, the sample mediums were then analyzed for their cytokine releases for TNF‐α and IL‐6 with an enzyme‐linked immunosorbent assay (ELISA).

Additionally, the changes in nitric oxide (NO) levels were determined with slight modifications in the overall approach. Briefly, 5 × 10^5^ cells/well of RAW 264.7 macrophages were seeded in 24‐well cell culture flasks and incubated overnight for attachment. The next day, 50, 150, and 500 μg/mL concentrations were introduced to the cells for 2 h as pre‐treatments and the mediums were discarded. Then, 0.5 μg/mL LPS‐containing mediums were given for an inflammation period of 24 h. The nitric oxide (NO) formation of the murine macrophages was determined by Griess assay afterwards according to Verdon et al. (1995).

### 2.10 Colonic inflammation model

The colonic inflammation model was adopted from Alyamac et al. (2021). CaCo‐2 cells were seeded in 12‐well plates and incubated for full confluency. Then, a 21‐day period was counted for differentiation by changing the cell culture medium every other day. On the 18th day, 2 × 10^8^ RAW 264.7 cells were seeded in a 75 cm^2^ cell culture flask (approximately 80% confluency). When the cells are attached overnight, a starvation period of 16 h started using 3% FBS‐containing medium. Then, LPS was introduced to the cell culture flask for a final concentration of 0.5 μg/mL for 12 h for the inflammatory response of macrophages. At the end of the inflammation period for macrophage cells, TNF‐α release of the inflammatory medium was controlled with an ELISA assay. As TNF‐α is one of the earliest and high‐concentration cytokines that are released in inflammatory conditions, it was selected as the marker cytokine for the confirmation of inflammation. The medium obtained by this was considered as the inflammatory medium (IM) to induce colonic inflammation. On the 21st day of differentiation of the CaCo‐2 enterocytes, GTE in the same concentrations as 50, 150, and 500 μg/mL as mixed samples were introduced as pre‐treatments for 4 h. Next, the pre‐treatment mediums were discarded, and IM medium obtained from the RAW 264.7 macrophages were given to the CaCo‐2 cells to induce colonic inflammation. The mediums were collected from each sample and their cytokine releases for IL‐6 and IL‐8 were analyzed with ELISA.

### 2.11 Statistical analyses

All experiments were conducted at least in triplicate (*n* ≥ 3) and the results were expressed as mean ± SEM. Statistical analyses of the data were performed by either two‐tailed two sample *t*‐tests or one‐way analysis of variance (ANOVA) comparing a sample with the experimental control (<.05). GraphPad Prism (version 8.0.2) software was used in preparing the figures and conducting the statistical analyses. Each statistical test applied for data evaluation was given in corresponding figure legends.

## 3 RESULTS AND DISCUSSION

### 3.1 Phytochemical composition and antiradical activity of the GTE

Natural compounds found in the plant‐based materials have long been studied for their phytochemical compositions and antiradical activities. Likewise, plants of Asteraceae have been reported to be important for food and pharmaceutical sciences due to their antioxidant potential (Bessada et al., 2015). Therefore, we started our investigation by determining some common phytochemical screening and antiradical capacity determinations. The total phenolic and flavonoid contents, as well as the antiradical activities in terms of TEAC, DPPH radical scavenging activity, and FRAP are given in Table 1. There was a similarity between the results of total phenolic and flavonoid contents as expected (Vermerris, 2006). The results of this study and the others vary due to raw material and extraction conditions. The phenolic compounds are secondary metabolites of plants in their defense mechanism. Therefore, they might differ according to the stress types and magnitudes that the plant was exposed to (Dixon & Paiva, 1995). It was stated that combining two or more solvents in the phenolic compound extraction step might result in various constituents when compared to a single solvent system due to polarity (Alara et al., 2021). Furthermore, there are two other publications reporting the antioxidant capacity of the *S. hispanicus* plant in literature (Berdja et al., 2021; Marmouzi et al., 2017). However, from the experimental point of view, variations in test method and extraction conditions of the test material cause difficulties in comparisons (Frankel & Meyer, 2000).

**TABLE 1:** Phytochemical composition and antiradical activities of the golden thistle hydromethanolic extract^a^.

Phytochemicals	Content^b^ (μmol/g sample)	Standard
Total phenolic content (μmol/g sample)	81.42 ± 5.69	Gallic acid
Flavonoid content (μmol/g sample)	10.09 ± 0.53	Catechin
	6.47 ± 0.32	Rutin

Antiradical activity	Content^b^ (μmol/mg sample)	Standard
TEAC (μmol/mg sample)	0.182 ± 0.008	Trolox
DPPH Radical Scavenging (μmol/mg sample)	0.102 ± 0.001	Trolox
FRAP (μmol/mg sample)	0.870 ± 0.11	Trolox
	0.73 ± 0.09	Ascorbic acid

^a^ The results are given as mean ± SD for each value.

^b^ Phytochemical compositions and antiradical activities are given as standard equivalents in the next column for the corresponding assay.

### 3.2 Chlorogenic acid bioavailability in CaCo‐2 cells

**FIGURE 1:**
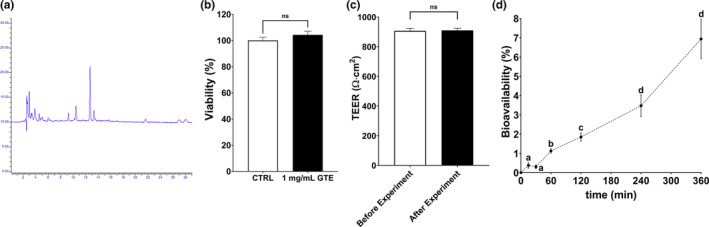
Chlorogenic acid bioavailability of GTE on CaCo‐2 cells in vitro. (a) Representative HPLC chromatogram of GTE sample, (b) cytotoxic effects of the 1 mg/mL GTE on CaCo‐2 cells, (c) TEER measurements before and after the bioavailability experiment, and (d) bioavailable CGA content passed through the basolateral part with respect to time. The results are given as mean ± SEM for at least three replicates (*n* ≥ 3). For sections (b) and (c), two sample *t*‐tests were applied (*p* < .05); and for section (d), one‐way ANOVA was used for each timepoint with Tukey's post hoc test. Different letters for the timepoints represent statistical significance (*p* < .05). CGA, chlorogenic acid; GTE, golden thistle extract; TEER, transepithelial electrical resistance.

Initially, we determined the phenolic compounds present in the GTE. According to the HPLC results, chlorogenic acid (5‐*o*‐caffeoylquinic acid, CGA) was found to be the abundant phenolic compound in the GTE (Figure 1a). There were some smaller peaks in the chromatogram, yet none of them could be matched with the other standard phenolic compounds. Then, investigating in vitro bioavailability of CGA was aimed.

We used CaCo‐2 cells on the bicameral transmembrane system because the utilization of differentiated CaCo‐2 cells for transport studies of phytochemical compounds has been well‐established (Iftikhar et al., 2020). We first confirmed the viability before the main investigations (Figure 1b). The MTT test showed that 1 mg/mL GTE did not exert cytotoxic activity as there were no difference in viability between GTE‐applied and non‐treated control cells (*p* > .05). It is also important to ensure that the differentiated CaCo‐2 enterocytes are tightly attached to prevent any leakages from the apical side. Thus, we measured TEER values before and after 6 h of bioavailability assay (Figure 1c). For both conditions, approximately 900 Ω cm^2^ TEER values were determined with no statistical difference prior to and after the assay (*p* > .05). The TEER values observed for our samples were higher than the accepted TEER value of 250 Ω cm^2^ (Volpe et al., 2007).

As the most abundant phenolic compound in the sample was detected as the CGA, we focused on the in vitro bioavailability of the CGA in the GTE. Then, 1 mg/mL GTE (containing 0.61 ± 0.16 mg/L CGA) was applied to the apical side of the membrane. Figure 1d shows the bioavailabilities of CGA with respect to time. The final CGA bioavailability was 6.94% after 6 h. During this assay, we also included a cell‐free transmembrane of the same GTE application and found that 19.69% of CGA bioavailability after 6 h (data not shown). In literature, similarly lower bioavailabilities were reported for CGA in 60 min in similar conditions (Dupas et al., 2006; Konishi & Kobayashi, 2004). Originating from the bioavailability results of Figure 3d, we concluded that the transfer trend could be assumed as linear, and the apparent permeability of CGA in our bioavailability model was applicable (Equation 1). The *P*_app_ of CGA in the GTE was calculated as (1.82 ± 0.009) × 10^−6^ cm/s, which is in accordance with the reported *P*_app_ of the CGA in *Flos Lonicerae Japonicae* reported by the same researchers (Zhou et al., 2015). CGA is a frequently found phenolic compound of plant‐based raw materials and is the abundant phenolic compound of the Asteraceae (Naveed et al., 2018). Although there are health attributes associated with the CGA, it is well known and confirmed once again that the bioavailability values are generally low for crude extracts and raw materials (Upadhyay & Mohan Rao, 2013; Velderrain‐Rodríguez et al., 2014). Therefore, further applications or product developments are needed to enhance the bioavailabilities of phenolic compounds for better achievements from a nutritional perspective.

### 3.3 Effects on starch digestibility

**FIGURE 2:**
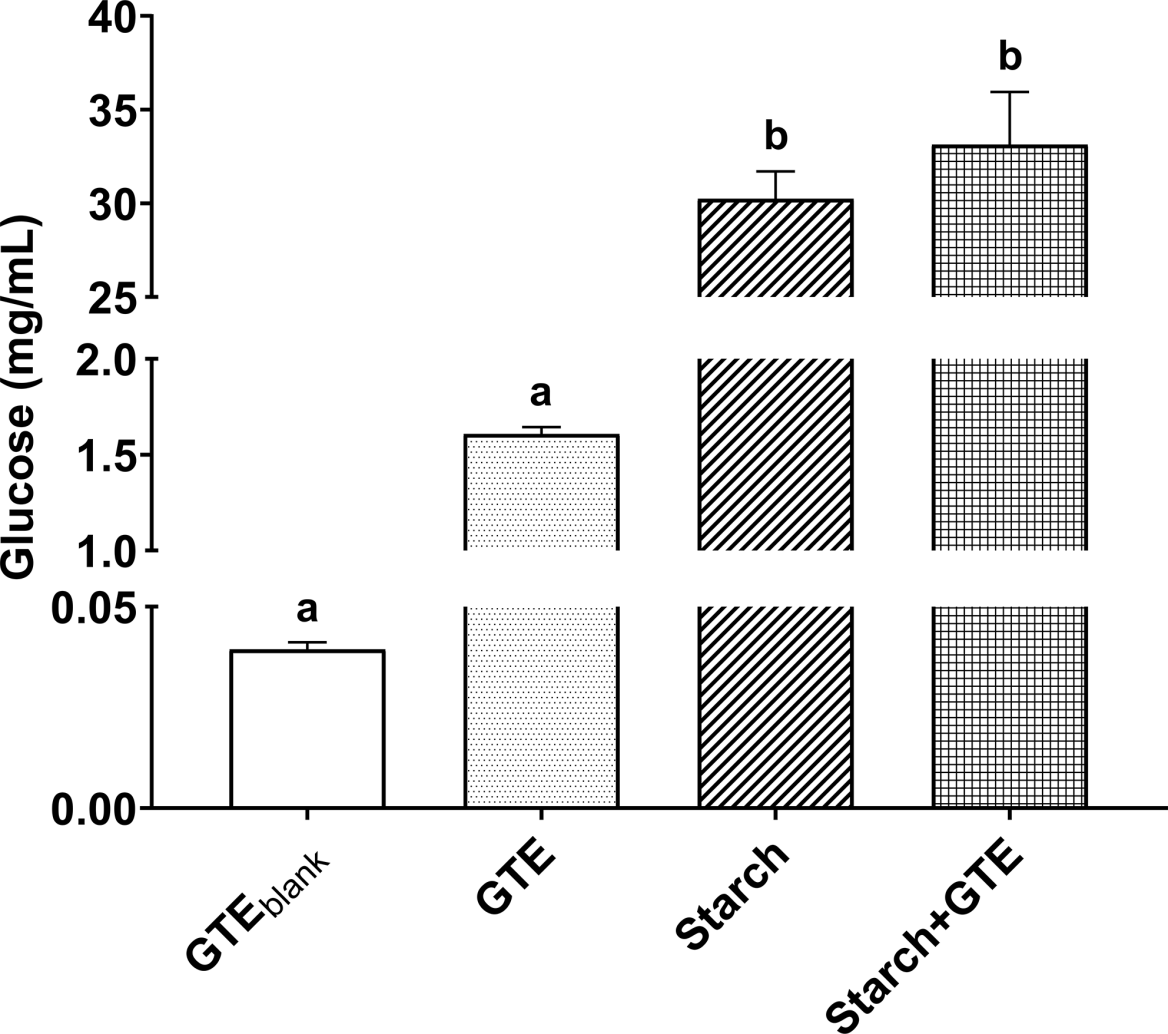
Effects of GTE on starch digestibility in test tube digestion. GTE_blank_: Experimental group in which no digestion procedure was applied, GTE: Digested GTE sample, Starch: Digested 1% starch solution, Starch + GTE: Digested sample of 1% starch solution and 1 mg/mL GTE combination. The results are given as mean ± SEM for three replicates (*n* = 3). Different letters on sample means represent statistical significance according to one‐way ANOVA with Tukey's post hoc test. GTE, golden thistle extract.

GTE was digested with a 1% starch solution to detect the possible effect on glucose release from digestion. However, we could not obtain any significant results (Figure 2). In the first group, the free glucose content in GTE was found to be close to 0.04 mg/mL. Additionally, we included a solely digested GTE to eliminate the possible release of glucose from the extract itself. Despite 1.61 mg/mL glucose was released as the result of in vitro digestion, there was no statistical significance between digested and undigested samples (*p* > .05). Although the experimental control group (Starch) released 18.8‐folds higher glucose than the GTE, as expected (*p* < .05), we found no difference when we incorporated 1 mg/mL GTE into a final concentration of digestion (*p* > .05). The inhibitory activity of GTE on mammalian glycosidases was already reported elsewhere (Marmouzi et al., 2017), yet we wanted to evaluate the glucose release activity under in vitro digestion conditions as a more complex medium. However, the starch digestibility approach did not result in significantly lower release of glucose when the starch solution is digested alongside with the GTE. Therefore, it can be concluded that GTE phytochemicals could not affect the digestive enzymes in our experimental conditions while mimicking human digestion.

### 3.4 Effects on glucose absorption through CaCo‐2 cells

**FIGURE 3:**
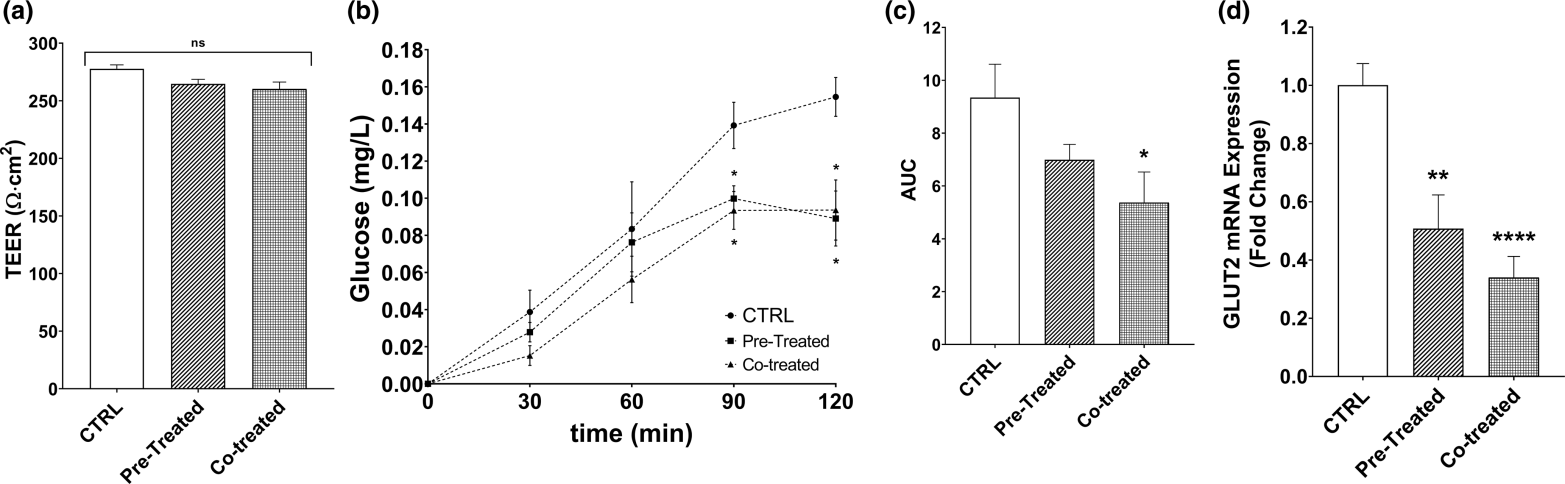
Effects of GTE on glucose efflux in CaCo‐2 cells. (a) TEER measurements of the experimental groups before the efflux study, (b) glucose contents in the basolateral side with respect to time, (c) area under curve values of the glucose efflux values determined in (c), and (d) relative GLUT2 mRNA expressions of the experimental groups of the cells in (c). CTRL: non‐treated control group, Pre‐treated: Experimental group in which 1 mg/mL GTE was applied as a pre‐treatment for 2 h, Co‐treated: Experimental group in which 1 mg/mL GTE and 25 mM glucose was introduced at the same time. The results are given as mean ± SEM for three replicates (*n* = 3). For statistical analyses, one‐way ANOVA was applied with Tukey's post hoc test. *p < .05, **p < .01, ****p < .0001. AUC, area under curve; CTRL, control; ns, non‐significant; TEER, transepithelial electrical resistance.

Plants of Asteraceae (Compositae), such as *Anacyclus pyrethrum* DC (Ardalani et al., 2021) and *Crassocephalum crepidioides* Benth. S. Moore (Bahar et al., 2017) have been reported to have antidiabetic activity. Thus, we also aimed to investigate whether GTE influenced glucose release and efflux as a model of in vitro antidiabetic activity (Figure 3). As 1 mg/mL GTE concentration was found to be nontoxic to the CaCo‐2 cells for 6 h (Figure 1b), same concentration was selected for the investigation of antidiabetic activity as well. Before the GTE or glucose introduction, TEER values were recorded as higher than 250 Ω cm^2^ in all experimental groups (*p* > .05, Figure 3a). Then, 1 mg/mL GTE was introduced to the apical side of the pre‐treated group for 2 h. After discarding GTE from the pre‐treated group, other experimental conditions were applied at the same time. Then, 25 mM glucose was given to the apical side of each group, however, in the co‐treated group, 1 mg/mL GTE was additionally given at the same time. The effluxed glucose content in the experimental control group, in which only 25 mM glucose application to the apical side was included, increased in every timepoint for 2 h (Figure 3b). Throughout the assay, the non‐treated negative control group was higher than the two GTE‐treated experimental groups. Yet, the differences became significant after 90 min (Figure 3b), as the pre‐ and co‐treated groups were 28.1% and 33.1% lower in glucose efflux when compared to the control, respectively (*p* < .05). At the end of the experiment, the control group had 0.15 mg/L glucose in the basolateral side, while both pre‐ and co‐treated groups had 0.09 mg/mL glucose. The differences between the experimental groups increased at the end of the experiment as the final glucose effluxes were 39.4% lower for pre‐, and 42.6% lower for co‐treated GTE samples (*p* < .05). We also calculated the total area under curve (AUC) values of the control, pre‐, and co‐treated GTE groups as 9.35, 6.99, and 5.37 values, respectively (Figure 3c). According to the results obtained, GTE co‐treated group had 42.5% less AUC than the control group (*p* < .05), while the AUC between the pre‐treated and control groups were insignificant (*p* > .05).

Relative GLUT2 mRNA expressions of the same cells of glucose efflux study were also determined (Figure 3d). The results showed that the relative mRNA expressions of GLUT2 were lower for both pre‐ and co‐treated cells (*p* < .05). Regarding the non‐treated control group as 100%, GLUT2 mRNA expressions of pre‐ and co‐treated cells were 49% and 66% lower, respectively. Therefore, it can be concluded that the GTE phytochemicals might be considered as a candidate for further studies regarding such indirect activity on antidiabetic research.

### 3.5 Effects on systemic inflammation markers

**FIGURE 4:**
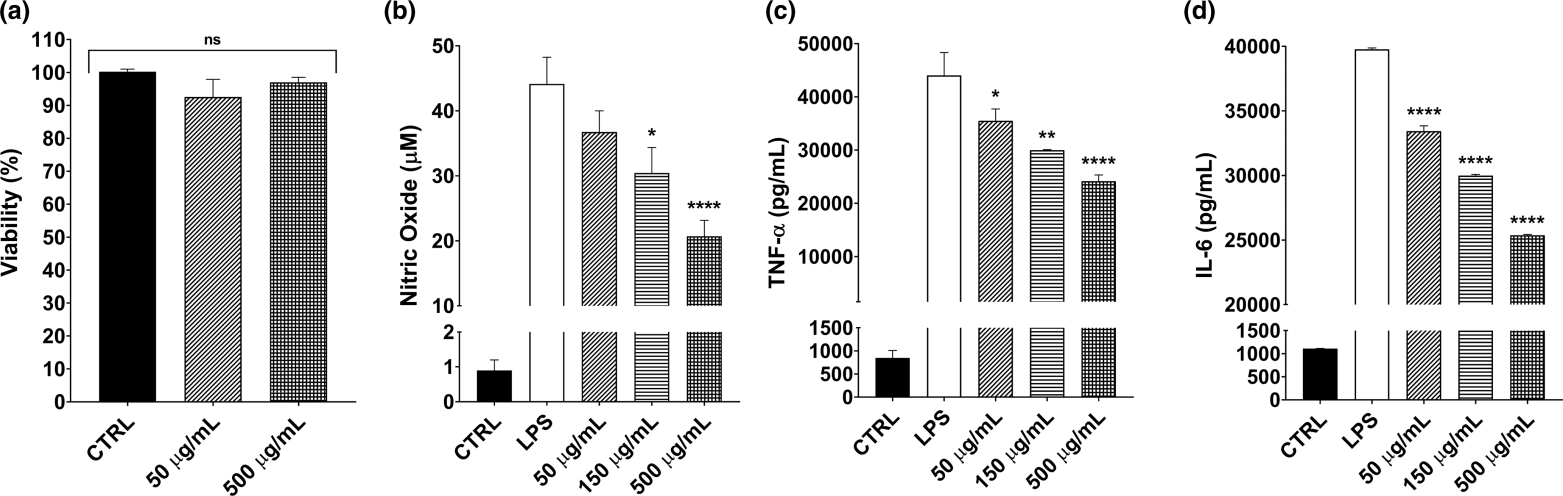
Effects of GTE on systemic inflammation model of RAW 264.7 cell line in vitro. (a) Cytotoxicity results of the lowest and the highest concentrations of extract mixture on RAW 264.7 mouse macrophages determined by an MTT test after 4 h exposure to the extract, (b) NO release after the RAW 264.7 cells were first pre‐treated with 50–500 μg/mL extract mixture as pre‐treatments for 2 h, followed by an application of 0.5 μg/mL LPS for 24 h, detected by Griess assay, (c) TNF‐α and (d) IL‐6 releases of RAW 264.7 cells after 2 h of pre‐treatments and the application of 0.5 μg/mL LPS for 12 h, determined by ELISA. The results are given as mean ± SEM for at least three independent experiments (*n* = 3). One‐way ANOVA was used in statistical analyses with Tukey's post hoc tests (*p < .05, **p < .01, ***p < .001, ****p < .0001). ANOVA, analysis of variance; ELISA, enzyme linked immunosorbent assay; IL‐6, interleukin 6; LPS, lipopolysaccharide; NO, nitric oxide; TNF‐α, tumor necrosis factor alpha.

In our research, another goal was to get an insight on the preventive effects of GTE against inflammation as the plants of Asteraceae, such as artichoke (*Cyanara scolymus*), chicory (*Cichorium intybus*), or dandelion species (*Taraxacum* spp.), are known for their suppressive effects on inflammatory markers (Rolnik & Olas, 2021). Furthermore, the golden thistle (*S. hispanicus* L.) reduced the TNF‐α, IL‐6, and some other inflammatory markers in phytohaemagglutin (PHA) induced peripheral blood mononuclear cells (PBMC) (Berdja et al., 2021; Kandil et al., 2020). We treated RAW 264.7 cells with low (50 μg/mL) and high (500 μg/mL) GTE and did not observe any cell toxicity (*p* > .05, Figure 4a). Then, we induced inflammation by 0.5 μg/mL LPS for 12 h. NO, TNF‐α and IL‐6 were selected as inflammatory markers and their levels were measured with or without GTE. The first parameter, nitric oxide (NO), is a free radical directly associated with macrophage activity (Butler & Williams, 1993). In the nitric oxide (NO) assay, we found that the LPS treatment elevated NO levels by 49‐folds (Figure 4b). However, there was a dose‐dependent decrease in NO levels when the cells were pre‐treated with different concentrations of GTE for 2 h. The NO concentrations were 36.5 ± 11.9, 31.4 ± 11.7, and 19.9 ± 8.4 μM for 50, 150, and 500 μg/mL pre‐treatments, respectively (Figure 4b). The results reported for RAW 264.7 macrophages treated with dandelion (*Taraxacum officinale*) (Park et al., 2011), and yarrow (*Achillea millefolium* L.) (Burk et al., 2010) extracts were in accordance with our results. Thus, the golden thistle might be another plant that can reduce the NO levels.

Although TNF‐α and IL‐6 are considered the primary mediators of inflammation in the body, the overproduction can lead to severe conditions (Neurath & Finotto, 2011; Strieter et al., 1993). According to the results obtained from ELISA, TNF‐α, and IL‐6 levels of RAW 264.7 cells were increased by LPS treatment (Figure 4c,d). When the macrophages were pre‐treated with 50, 150, and 500 μg/mL of concentrations for 2 h, the TNF‐α levels reduced to 35.47 ± 4.5, 29.99 ± 0.2, and 24.15 ± 2.3 ng/mL, respectively, (*p* < .05). The reductions were in 19%–45% range. Furthermore, GTE treatment significantly reduced IL‐6 levels. IL‐6 levels of the pre‐treated samples were detected as 33.43 ± 0.22, 29.99 ± 0.16, 25.36 ± 0.13 ng/mL in increasing concentrations. The reductions were 15.9% for 50 μg/mL, 24.6% for 150 μg/mL, and 36.2% for 500 μg/mL concentrations. Our results showed that the GTE reduced the release of both cytokines. The findings in our study are similar to the plants such as *Ageratina pihinchensis*, *Artemisia halodendron*, and *Artemisia montana* in the inflammatory cytokine release trends (Jeong et al., 2018; Jin et al., 2019; Sánchez‐Ramos et al., 2018).

### 3.6 Effects on colonic inflammation markers

The colon tissue is exposed to different immune‐related factors, including toxins, microorganisms, and foreign antigens. Thus, regulation of the immune system is essential for colonic cells. In the colonic inflammation model, the CaCo‐2 cell line was chosen due to its ability to mimic the intestinal system after a certain period of differentiation, alongside with macrophages. LPS‐treated RAW 264.7 cells were used to mimic systemic inflammation and we obtained inflammatory medium (IM) from RAW 264.7 cells. We treated RAW 264.7 cells with 0.5 μg/mL LPS for 12 h. Then, the TNF‐α cytokine level was measured in the inflammatory medium of RAW 264.7 cells. There was a 178‐folds difference between the LPS treated (27.536 ng/mL) and non‐treated (0.154 ng/mL) cell culture mediums for TNF‐α.

**FIGURE 5:**
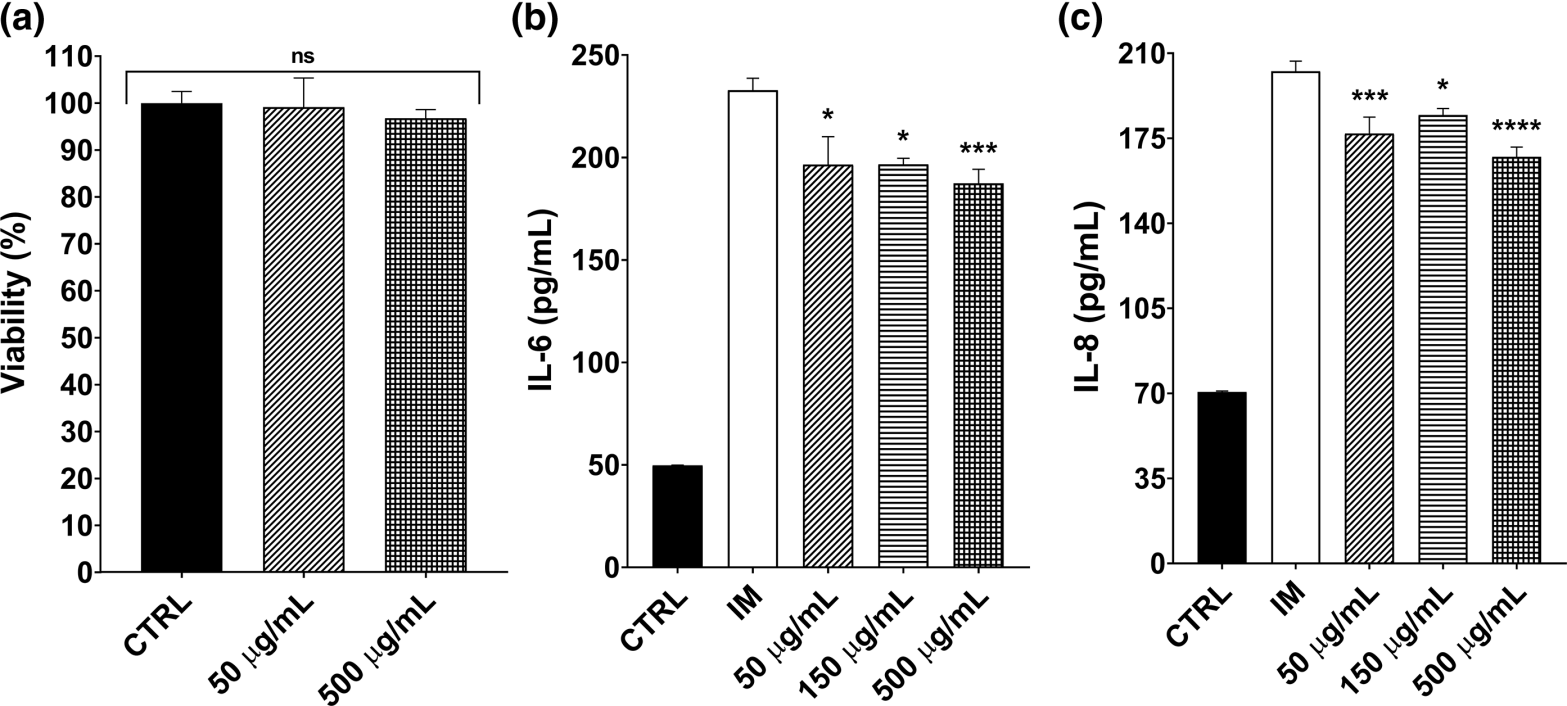
Effects of GTE on colonic inflammation using RAW 264.7 and CaCo‐2 lines in vitro. (a) Cytotoxicity results of the lowest and the highest concentrations of extract mixture for 4 h on CaCo‐2 cells determined by an MTT test, (b) IL‐6 and (c) IL‐8 releases of CaCo‐2 cells after 4 h of pre‐treatments and the application of IM for 12 and 24 h. Samples for IL‐6 and IL‐8 were taken at the end of 24 and 12 h, respectively. The cytokine releases were detected by ELISA. The results are given as mean ± SEM for at least three independent experiments (*n* = 3). One‐way ANOVA was used in statistical analyses with Tukey's post hoc tests (*p < .05, **p < .01, ***p < .001, ****p < .0001). ANOVA, analysis of variance; ELISA, enzyme linked immunosorbent assay; IL, interleukin.

Additionally, we used low (50 μg/mL) and high (500 μg/mL) concentrations of GTE to test any possible cytotoxicity on Caco‐2 cells and found no significant cytotoxicity with an MTT assay (Figure 5a, *p* > .05). In the colonic inflammation model, the CaCo‐2 cells were pre‐treated with 50–500 μg/mL range of GTE for 4 h. Then, these pre‐treatment mediums were discarded, and the prepared IM was introduced to the cells as the inducer of colonic inflammation. Samples were taken at the end of 12 and 24 h, for the detection of IL‐8 and IL‐6 cytokine releases with ELISA, respectively. The first two concentrations of 50 and 150 μg/mL showed 15.6 and 15.5% IL‐6 reduction, respectively, (*p* < .05, Figure 5b). Furthermore, 500 μg/mL GTE reduced the IL‐6 release by 19.5% (*p* < .05). When the cell culture medium was collected after 12 h, the IL‐8 content of the non‐treated control group was 65.2% lower than that of the IM group (*p* < .05). The reductions in IL‐8 were not again in a dose‐dependent manner, in a range of 167.35–184.57 pg/mL (Figure 5c). Overall, the GTE mixture concentrations were significantly lower than the IM control groups (*p* < .05). IL‐6 is a pro‐inflammatory cytokine released in the earlier stages of most inflammation cases, while IL‐8 is an inflammatory cytokine important in colonic inflammation and the patients with IBD (Daig et al., 1996; Grimm et al., 1996). In literature, plants like *Rhanterium suaveolens* or chamomile reduced both IL‐6 and IL‐8 levels in CaCo‐2 and HT‐29 cell lines, respectively (Chelly et al., 2021; Kogiannou et al., 2013). The GTE might be another addition of these Asteraceae plants for the studies of colonic inflammation.

## 4 CONCLUSION

We screened possible physiological bioactivities of *S. hispanicus* hydromethanolic extracts in the study. The overall phytochemical bioavailability of the golden thistle crude extract was relatively lower. However, applied concentrations in the glucose efflux and inflammation models showed promising effects in vitro. One limitation of our study was that we tested the biological activity of GT crude extract in our experimental models. However, it might be important to target a molecule or molecule group that would have an effect on glucose efflux and inflammation. Therefore, further studies can be provided by the identification and isolation of potential bioactive molecules to be used in food and pharmaceutical developments after in vivo trials.

## AUTHOR CONTRIBUTIONS

**Cansu Ozel‐Tasci:** Formal analysis (lead); investigation (lead); methodology (equal); writing – original draft (lead). **Sukru Gulec:** Conceptualization (lead); funding acquisition (lead); methodology (equal); project administration (lead); resources (lead); supervision (lead); writing – review and editing (lead).

## CONFLICT OF INTEREST STATEMENT

The authors declare no conflict of interest.

## Data Availability

The data that support the findings of this study are available from the corresponding author upon reasonable request.
